# Prevalence of malnutrition and its associated factors among 18,503 Chinese children aged 3–14 years

**DOI:** 10.3389/fnut.2023.1228799

**Published:** 2023-12-11

**Authors:** Xiaoqian Zhang, Qiong Wang, Ziyu Gao, Zifeng Zhang, Jing Wu, Zhixin Zhang, Wenquan Niu

**Affiliations:** ^1^Graduate School, Beijing University of Chinese Medicine, Beijing, China; ^2^Department of Pediatrics, China-Japan Friendship Hospital, Beijing, China; ^3^Center for Evidence-Based Medicine, Capital Institute of Pediatrics, Beijing, China; ^4^International Medical Services, China-Japan Friendship Hospital, Beijing, China

**Keywords:** malnutrition, children, prevalence, risk factor, association

## Abstract

**Background:**

Child malnutrition places a major burden on global public health. We aimed to estimate the prevalence of child malnutrition and identify its potential factors among children aged 3–14 years from Beijing and Tangshan.

**Methods:**

We cross-sectionally recruited 18,503 children aged 3–14 years from September 2020 to January 2022, according to a stratified cluster random sampling strategy. Child malnutrition was defined according to the World Health Organization criteria. Data were analyzed by STATA software and R language.

**Results:**

The prevalence of malnutrition among 18,503 children was 10.93%. After multivariable adjustment, seven factors significantly associated with child malnutrition were parental education (adjusted odds ratio, 95% confidence interval, *p*: 1.52, 1.40 to 1.67, <0.001), family income (1.23, 1.16 to 1.30, <0.001), fast food intake frequency (1.14, 1.06 to 1.21, <0.001), night meals intake frequency (1.09, 1.04 to 1.15, <0.001), eating speed (1.01, 1.01 to 1.02, <0.001), maternal obesity (0.97, 0.95 to 0.99, <0.001), and paternal obesity (0.97, 0.96 to 0.98, <0.001). The seven significant factors had better prediction performance (area under the receiver operating characteristic, 0.956) for child malnutrition.

**Conclusion:**

Approximately 10% of Chinese children aged 3–14 years were in malnutrition status, and seven factors were found to be significant predictors for child malnutrition.

## Introduction

Child malnutrition, including stunting, wasting, and being underweight, is featured by inadequate or unbalanced intake of protein energy or nutrients, and it represents a public health issue, deserving special awareness (1). Statistics from the fund of the United Nations Children (UNICEF) and World Health Organization (WHO) show that 22.0% and 6.7% of children younger than 5 years were separately affected by stunting and wasting worldwide (2). In China, the prevalence of underweight and stunting in children younger than 5 years was estimated to be 3.6 and 9.9%, respectively (3), and the prevalence of malnutrition in 7–18-year-old children was 8.6% (4). The detrimental impact of child malnutrition is manifold because it can not only threaten survival, development, and growth of children but also undermine economic growth and national development (5). The causes of child malnutrition are complex, and no consensus has thus far been reached on its risk profiling.

It is widely accepted that besides factors relating to inheritance and diseases, child malnutrition is mainly caused by inadequate nutrient intake. Over the past decades, dietary patterns and modern lifestyles have undergone profound changes, along with the intensification of industrialization and urbanization in China (6), and we are experiencing significant public health challenges (7). Due to inadequate intake of vegetables and fruits, as well as excessive intake of red meat, oil, and salt, nutrition deficiency and nutrition imbalance have aroused extensive concerns (8, 9). There is evidence for the significant influence of parental dieting behavior affected by rhythm of life, family income and education on child dieting behavior, and weight status (10). Hence, identifying potential factors underlying child malnutrition to facilitate the development of personalized intervention and follow-up care is urgently needed. Some factors, such as parental obesity and eating habits, were reported to be associated with child malnutrition (11–13), whereas the majority of these association studies focused on only a few factors and were largely underpowered to provide reliable estimates.

To fill this gap in knowledge, a large cross-sectional survey of children of 3–14 years of age from Beijing and Tangshan estimated the prevalence of child malnutrition and identified factors that are independently associated with the risk of child malnutrition.

## Methods

### Study design

We conducted two cross-sectional surveys from September 2020 to January 2022. The first survey was performed in Beijing and Tangshan (in Hebei province) from September to December 2020, and the second in January 2022 in Pinggu district, Beijing.

According to the 2022 census data, there are approximately 21.8 million persons in Beijing and 7.7 million persons in Tangshan. There are 16 districts in Beijing and 7 districts in Tangshan. Beijing covers an area of 16,410 km^2^, and Tangshan covers an area of 13,472 km^2^. *Per capita* disposable income was 77,400 RMB in Beijing and 39,600 RMB in Tangshan.

### Study participants

The selection process of study participants was reported previously (14, 15). Specifically, in the first survey, study participants were consisted of preschool-aged children attending junior to senior kindergarten classes. We randomly selected 4 out of 16 districts in Beijing and 2 out of 7 districts in Tangshan by using the stratified and cluster sampling methods. In total, 5 kindergartens were selected from each district, and 30 kindergartens were included finally. In the second survey, study participants included children attending primary school or junior high school. We randomly selected 26 schools, including 8 primary schools and 18 junior high schools, in Pinggu district, Beijing. In this study, data were collected from children aged 3–14 years in 30 kindergartens and 26 schools in Beijing and Tangshan.

### Data collection

We collected data by self-designed questionnaires. To ensure reliability and validity, both questionnaires were separately distributed in 200 samples before formal circulation, and the reliability coefficient (alpha) was over 0.85.

We integrated the items from both questionnaires, covering four main areas as follows: (i) demographic area: age, sex, nationality, date of birth, height, weight, and food and drug allergy; (ii) fetal and neonatal area: birth body length, gestational age, delivery mode, pregnancy order, delivery order, assisted reproduction, twin birth, infancy feeding, breastfeeding duration, and time of adding solid food; (iii) lifestyle-related area: sitting time, screen time, outdoor activity time, sleep duration, fall asleep time, eating speed, number of dental caries, and weekly intake frequencies of sweet food, night meals, and fast food; (iv) family-related area: parental age, parental weight, parental height, parental education, and family income. Body mass index (BMI) was calculated as weight divided by height squared (kg/m^2^).

The questionnaires were generated by the “Wenjuanxing” website.^1^ As an online platform in China, the “Wenjuanxing” can yield a unique QR code for each questionnaire. The QR code can be recognized by smartphones and was sent to the parents or guardians by teachers-in-charge via the “WeChat” social media application. The “Wenjuanxing” platform automatically integrated data from individual questionnaires as an Excel spreadsheet, which can be downloaded for analysis.

### Quality control

Before the distribution of questionnaires, healthcare physicians and teachers-in-charge selected in this survey from all schools and kindergartens were trained to be familiar with the survey procedures and each item in the questionnaires. During the survey, healthcare physicians and teachers-in-charge can help parents or guardians of participating children to fill out questionnaires. At the end of the survey, data were downloaded into a Microsoft Office Excel™ spreadsheet from the “Wenjuanxing” platform and were strictly checked by our trained staff. Healthcare physicians and teachers-in-charge were requested to contact the parents or guardians of participating children to resupply or confirm information that was obviously abnormal in the questionnaires. Body weight (to the nearest 0.1 kg) and height (to the nearest 0.1 cm) were measured by healthcare physicians.

### Definitions of stunting, underweight, wasting, and malnutrition

Child malnutrition included stunting, underweight, and wasting. In this study, we adopted the WHO criteria to define child malnutrition. Specifically, the 2006 WHO Child Growth Standard (16) defined stunting, underweight, and wasting for children aged 0–5 years (0–60 months), and the 2007 WHO growth reference for school-aged children and adolescents (17) defined stunting and wasting for children aged 5–19 years (61–228 months). The nutritional status of a child was evaluated by the Z-score, which was calculated using the deviation of the value of a child from the median of the reference population, divided by the standard deviation of the reference population. According to the WHO criteria, we defined stunting, underweight, and wasting based on two age stages: (i) children aged 36–60 months: stunting was defined as a height-for-age Z-score (HAZ) less than −2 standard deviations (SDs), underweight as weight-for-age Z-score (WAZ) of less than −2 SDs, and wasting as weight-for-height Z-score (WHZ) less than −2 SDs; (ii) children aged 61–180 months: stunting was defined as a height-for-age Z-score (HAZ) less than −2 standard deviations (SDs) and BMI for age Z-score (BMIZ) less than −2 SDs.

### Definitions of other items

Delivery modes included vaginal delivery and cesarean section. Gestational age was recorded in months, and birth between 37 and 42 gestational weeks was regarded as full-term birth. Birth body length (to the nearest 0.1 cm) was reported by the parents or guardians of participating children. Pregnancy order and delivery order meant the times of pregnancy and bearing birth, respectively.

Infancy feeding included pure breastfeeding, partial breastfeeding, and non-breastfeeding. Breastfeeding duration and time of adding solid food were recorded in months. Some lifestyle-related factors, including sitting time, screen time, and outdoor activity time recorded in hours, were calculated as the sum of time spent on workdays × 5 and weekends × 2 divided by 7. In this survey, sitting time included screen time, and screen time referred to the amount of time spent on screen-related behaviors such as watching TV and playing computer games (18). Sleep duration included time for a lunch break. Eating speed was calculated as the average of breakfast, lunch, and dinner. Sweet food refers to food with sweet taste (such as bread cakes and desserts), and fast food is defined as food with high energy and low nutrition, such as hamburger and pizza. Night meal refers to food eaten within 2 h before bedding. We investigated the weekly intake frequency, which was classified as every day, often (3–5 times), occasional (1–2 times), and none, of sweet food, fast food, and night meals.

Parental BMI was calculated from self-reported weight and height. Family education referred to the highest educational level of parents and was categorized as master’s degree or above, bachelor’s degree, and high school degree or below. Family income (RMB per year) was categorized as ≥300,000, 100,000–300,000, and < 100,000.

### Statistical analyses

Data were analyzed using STATA software version 16.0 (Stata Corp, College Station, TX, United States) and R programming environment (version 3.5.2). Study power was calculated using PS (Power and Sample Size Calculations) software version 3.0.

We assign values to categorical variables, as shown in Supplementary Table S1. The possible biases arising from different kindergartens and schools were assessed by the intraclass correlation coefficient (ICC). In theory, ICC can be used to quantify observed differences within and between clusters (19).

To reduce the influence of potential bias and confounding, we adopted 1:4 propensity score matching (PSM) between the malnutrition and control groups. Continuous variables are expressed as mean (standard deviation), in the case of no deviation from normal distribution based on the Skewness and Kurtosis tests, and as median (interquartile range). Categorical variables are expressed as count (percent). Between-group comparisons of variables were performed using the t-test, χ^2^ test, or rank-sum test, where appropriate. The variance inflation factor (VIF) was calculated to assess multiple collinearities.

To identify statistically significant risk factors for child malnutrition, logistic regression analyses were conducted before and after considering age, sex, twins and infancy feeding, birth length, pregnancy order, and delivery order. Effect-size estimates are expressed as odds ratio (OR) and 95% confidence interval (CI).

After adding significant factors associated with child malnutrition to the basic model, prediction performance was appraised from both calibration and discrimination aspects. From the calibration aspect, Akaike information criterion (AIC) and Bayesian information criterion (BIC) were used to observe how closely the prediction probability, by adding significant factors, reflected the actual observed risk and global fit of the modified risk model, and the calibration curves for different models provide another form of visualization. From the discrimination aspect, the receiver operating characteristic (ROC) curve of both models was also presented to observe the discrimination capability of identified significant factors. The net benefits of this addition were also inspected by decision curve analysis (DCA).

## Results

### Baseline characteristics

In the first survey from September to December 2020, questionnaires were distributed to the parents or guardians of 10,441 children. In the second survey in January 2022, 11,633 questionnaires were sent. We pooled data from both surveys and strictly reviewed the validity of the survey data. Finally, 18,503 questionnaires were deemed eligible for inclusion. In this study, the prevalence of child malnutrition was 10.93% (*n* = 2022), and stunting, underweight, and wasting were 1.34% (*n* = 248), 11.22% (*n* = 563), and 9.77% (*n* = 1808), respectively.

The baseline characteristics of participating children are shown in Table 1 and Supplementary Table S2. The ICCs for all factors under the study were all relatively low (<0.01), indicating a low probability of clustering within kindergartens or schools and a lower likelihood of differences in surveyed items (Supplementary Table S3).

**Table 1:** Baseline characteristics of study children.

Characteristics	Unmatched population			1:4 matched population		
	Children without malnutrition	Children with malnutrition		Children without malnutrition	Children with malnutrition	
	(*n* = 16,481)	(*n* = 2022)	*p*	(*n* = 8,088)	(*n* = 2022)	*p*
Demographic information						
Age (years)	7.92 (4.91, 11.17)	5.5 (4.33, 7.58)	<0.001	5.5 (4.33, 7.5)	5.5 (4.33, 7.58)	0.771
Boys	8,486 (51.5)	1,036 (51.2)	0.848	4,234 (52.3)	1,036 (51.2)	0.370
Nationality			<0.001			0.093
Han	15,368 (93.2)	1828 (93.4)		7,407 (91.6)	1828 (90.4)	
Others	1,113 (6.8)	194 (9.6)		681 (8.4)	194 (9.6)	
Height (cm)	132 (111, 153)	115 (105, 125)	<0.001	115 (106, 130)	115 (105, 125)	<0.001
Weight (kg)	29 (20, 46.5)	14 (10.5, 20)	<0.001	20.5 (17.5, 29)	14 (10.5, 20)	<0.001
BMI (kg/m^2^)	16.91 (15.18, 20.2)	10.68 (8.5, 12.86)	<0.001	16 (14.83, 18.01)	10.68 (8.5, 12.86)	<0.001
Food allergy	1,658 (10.1)	239 (11.8)	0.015	848 (10.5)	239 (11.8)	0.083
Drug allergy	694 (4.2)	81 (4.0)	0.707	312 (3.9)	81 (4.0)	0.758
Stunting	0 (0.0)	248 (12.3)	<0.001	0 (0)	248 (12.3)	<0.001
Underweight	0 (0, 0)	563 (67.7)	<0.001	0 (0)	563 (67.7)	<0.001
Wasting	0 (0.0)	1808 (89.4)	<0.001	0 (0)	1808 (89.4)	<0.001
Fetal and neonatal factors						
Birth length (cm)	50 (50, 52)	50 (50, 52)	0.174	50 (50, 52)	50 (50, 52)	0.013
Full-term birth (weeks)	13, 652 (90.10)	1729 (89.4)	0.346	767 (10)	205 (10.6)	0.411
Delivery mode			0.081			0.360
Vaginal delivery	8,125 (49.3)	1,049 (51.9)		4,080 (50.4)	1,043 (51.6)	
Cesarean section	8,356 (50.7)	973 (48.1)		4,008 (49.6)	979 (48.4)	
Pregnancy order	1 (1, 2)	2 (1, 2)	<0.001	1 (1, 2)	2 (1, 2)	0.005
Delivery order	1 (1, 2)	1 (1, 2)	<0.001	1 (1, 2)	1 (1, 2)	<0.001
Assisted reproduction	288 (1.7)	51 (2.5)	0.018	158 (2)	51 (2.5)	0.108
Twins	402 (2.4)	43 (2.1)	0.430	203 (2.5)	43 (2.1)	0.317
Infancy feeding			0.069			0.006
Pure breastfeeding	9,307 (56.5)	1,194 (59.1)		4,526 (56.0)	1,194 (59.1)	
Partial breastfeeding	5,688 (34.5)	665 (32.9)		2,968 (36.7)	665 (32.9)	
Non-breastfeeding	1,486 (9.0)	163 (8.1)		594 (7.3)	163 (8.1)	
Breastfeeding duration (months)	12 (0, 15)	12 (6, 18)	<0.001	12 (6, 18)	12 (6, 18)	0.906
Solid food introduction (months)	6 (6, 7)	6 (6, 7)	0.140	6 (6, 7)	6 (6, 7)	0.453
Lifestyle-related factors						
Outdoor activities (hours per day)	1.29 (1, 2)	1.29 (1, 2.29)	<0.001	1.43 (1, 2.29)	1.29 (1, 2.29)	0.412
Screen time (hours per day)	1.14 (0.64, 1.57)	1 (0.64, 1.57)	<0.001	1 (0.64, 1.57)	1 (0.64, 1.57)	0.014
Fall asleep time (hours per day)	9.5 (9, 10)	9 (9, 10)	<0.001	9 (9, 10)	9 (9, 10)	0.449
Sleep duration (hours per day)	9.29 (8.29, 10)	9.36 (8.64, 10.29)	<0.001	9.43 (8.57, 10.29)	9.36 (8.64, 10.29)	0.859
Eating speed (minutes)	16.67 (13.33, 21.67)	18.33 (15, 25)	<0.001	18.33 (13.33, 23.33)	18.33 (15, 25)	<0.001
Fast food intake frequency			<0.001			0.001
Every day	4,362 (26.5)	237 (11.7)		1,006 (12.4)	237 (11.7)	
3–5 times weekly	4,801 (29.1)	237 (11.7)		1,205 (14.9)	237 (11.7)	
1–2 times weekly	2,694 (16.3)	536 (26.5)		2021 (25.0)	536 (26.5)	
None or once in a while	4,624 (28.1)	1,012 (50.1)		3,856 (47.7)	1,012 (50.1)	
Sweet food intake frequency			<0.001			0.045
Every day	2,348 (14.2)	202 (10.0)		817 (10.1)	202 (10.0)	
3–5 times weekly	7,314 (44.4)	627 (31.0)		2,742 (33.9)	627 (31.0)	
1–2 times weekly	5,356 (32.5)	957 (47.3)		3,564 (44.1)	957 (47.3)	
None or once in a while	1,463 (8.9)	236 (11.7)		965 (11.9)	236 (11.7)	
Night meals intake frequency			<0.001			0.012
Every day	5,691 (34.5)	356 (17.6)		1,682 (20.8)	356 (17.6)	
3–5 times weekly	3,417 (20.7)	285 (14.1)		1,152 (14.2)	285 (14.1)	
1–2 times weekly	2,540 (15.4)	412 (20.4)		1,582 (19.6)	412 (20.4)	
None or once in a while	4,833 (29.4)	969 (47.9)		3,672 (45.4)	969 (47.9)	
Dental caries	0.00 (0.00, 2.00)	1.00 (0.00, 2.00)	0.318	1.00 (0.00, 2.00)	1.00 (0.00, 2.00)	0.343
Family-related factors						
Maternal age (years)	36 (33, 39)	35 (32, 38)	<0.001	35 (32, 38)	35 (32, 38)	<0.001
Paternal age (years)	37 (34, 40)	37 (33, 40)	<0.001	36 (33, 39)	37 (33, 40)	<0.001
Family education			<0.001			<0.001
High school degree or below	6,677 (40.5)	689 (34.1)		3,143 (38.9)	689 (34.1)	
Bachelor’s degree	9,039 (54.8)	1,019 (50.4)		4,399 (54.4)	1,019 (50.4)	
Master’s degree or above	765 (4.7)	314 (15.5)		546 (6.7)	314 (15.5)	
Family income (RMB per year)			<0.001			<0.001
<100, 000	6,964 (42.3)	731 (36.2)		3,273 (40.5)	731 (36.2)	
100, 000–300, 000	7,161 (43.5)	748 (37.0)		3,325 (41.1)	748 (37.0)	
>300, 000	2,356 (14.2)	543 (26.8)		1,490 (18.4)	543 (26.8)	
Maternal BMI	22.89 (20.76, 25.71)	22.04 (20.20, 24.51)	<0.001	22.60 (20.57, 25.22)	22.04 (20.20, 24.51)	<0.001
Paternal BMI	25.83 (23.46, 28.41)	25.06 (22.64, 27.76)	<0.001	25.57 (23.32, 28.34)	25.06 (22.64, 27.76)	<0.001

Data are expressed as median (interquartile range) or count (percent). *p*-value was calculated by the rank-sum test or the χ^2^ test, where appropriate. BMI, body mass index.

### Identification of potential factors for child malnutrition

Multiple collinearities revealed by variance inflation factor (VIF) indicated that no multiple collinearities existed between different variables (Supplementary Table S4).

As shown in Table 1, baseline variables were selected based on clinical relevance or with a *p*-value of less than 0.05 on univariate analyses. After adjusting for age, gender, twins and infancy feeding, birth length, pregnancy order, and delivery order, seven factors, namely, family education (OR, 95% CI, *p*: 1.52, 1.40 to 1.67, <0.001), family income (1.23, 1.16 to 1.30, <0.001), fast food intake frequency (1.14, 1.06 to 1.21, <0.001), night meals intake frequency (1.09, 1.04 to 1.15, <0.001), eating speed (1.01, 1.01 to 1.02, <0.001), maternal BMI (0.97, 0.95 to 0.99, <0.001), and paternal BMI (0.97, 0.96 to 0.98, <0.001) were found to be associated with the significant risk of child malnutrition, as shown in Table 2.

**Table 2:** Identification of potential factors for child malnutrition.

Significant variables	Univariate model (unadjusted)			Multivariable adjusted model^*^		
	OR	95% CI	*p*	OR	95% CI	*p*
Family education	1.43	1.32 to 1.54	<0.001	1.52	1.40 to 1.67	<0.001
Family income	1.23	1.17 to 1.30	<0.001	1.23	1.16 to 1.30	<0.001
Fast food intake frequency	1.07	1.02 to 1.12	0.008	1.14	1.06 to 1.21	<0.001
Night meals intake frequency	1.07	1.02 to 1.11	0.002	1.09	1.04 to 1.15	<0.001
Eating speed	1.01	1.01 to 1.02	<0.001	1.01	1.01 to 1.02	<0.001
Maternal BMI	0.98	0.97 to 0.99	<0.001	0.97	0.95 to 0.99	<0.001
Paternal BMI	0.97	0.96 to 0.98	<0.001	0.97	0.96 to 0.98	<0.001

BMI, body mass index; OR, odds ratio; 95% CI, 95% confidence interval. ^*^The *p*-value was calculated after adjusting for age, gender, twins and infancy feeding, birth length, pregnancy order, and delivery order.

The power to detect significance when estimating the risk for child malnutrition was over 80% for the above comparisons.

### Prediction performance assessment

**Figure 1:**
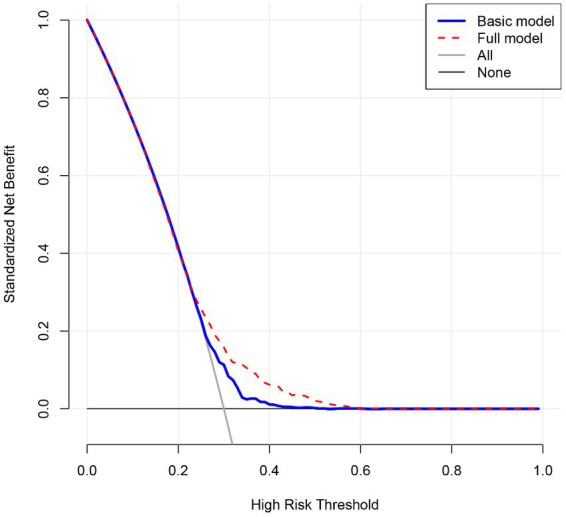
Decision curve analysis on the net benefits gained by adding seven significant factors associated with child malnutrition. Basic model included all variables under study with the exception of seven significant factors is presented in Table 2, and full model included all variables under the study.

We constructed both the basic model and full models to assess the prediction performance of seven significant factors identified above. The full model included all variables, and the basic model included all variables with the exception of seven significant factors. The prediction performance of both models was assessed from calibration and discrimination aspects, and the difference between the basic model and full model was compared. Significant improvement was observed in the prediction accuracy of the full model relative to that of the basic model (Table 3). In addition, the DCA plot showed that the net benefits gained by adding seven significant factors to the basic model were obvious (Figure 1).

**Table 3:** Prediction performance before and after adding seven significant factors identified for child malnutrition.

Statistics	Basic model	Full model
Calibration		
AIC	10050.156	3860.350
BIC	10215.485	4125.173
Discrimination		
AUROC	0.564	0.907
*p* value for AUROC	<0.001	

AIC, Akaike information criterion; BIC, Bayesian information criterion; AUROC, area under the receiver operating characteristic. Basic model included all variables under the study with the exception of seven significant factors is presented in Table 2, and full model included all variables under the study.

**Figure 2:**
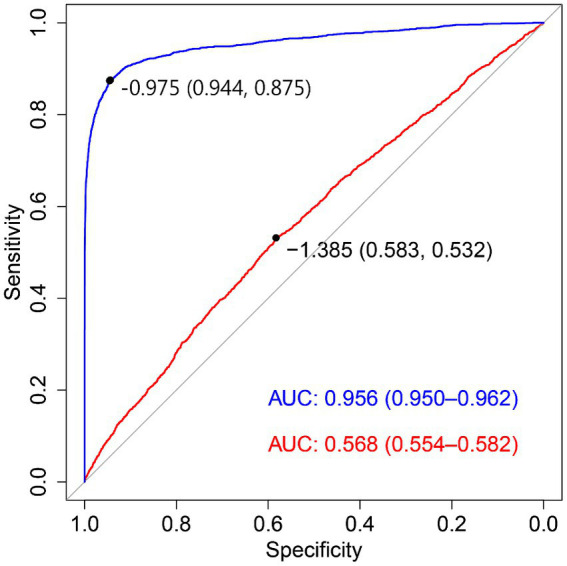
Receiver operating characteristic (ROC) curve for both basic and full models. AUC: area under curve. The red solid line corresponds to basic model, and the blue solid line corresponds to full model. Basic model included all variables with the exception of seven significant factors is presented in Table 2, and full model included all variables under the study.

The ROC curves are shown in Figure 2, and calibration curves are shown in Supplementary Figure S1. As revealed by the area under the receiver operating characteristic (AUROC) curve, both models differed significantly in discrimination (*p* < 0.001).

## Discussion

In this study, we aimed to estimate the prevalence of child malnutrition and identify its potential factors among 18,503 Chinese children aged 3–14 years from Beijing and Tangshan. The key finding of this study was that approximately one in ten children suffered from malnutrition in North China. Moreover, parental education, family income, fast food intake frequency, night meals intake frequency, eating speed, and parental obesity were significantly and independently associated with child malnutrition under the WHO criteria. Thus far, to the best of our knowledge, this is the first study that has explored the risk profiling of child malnutrition among Chinese preschool and school-age children.

Recently, the overall detection rate of malnutrition has shown a downward trend (4, 20, 21). As reflected in this study, approximately 10% of Chinese children aged 3–14 years was in malnutrition status (stunting: 1.34%, underweight: 11.22%, and wasting: 9.77%), which was higher than that released in 2019 among Chinese children aged 7–18 years at 8.64% (4). The difference in the prevalence can be attributable to the differences in diagnostic criteria and participant characteristics. In this study, for the sake of extrapolation and comparisons, we adopted the WHO criteria, instead of the China criteria (22). In addition to malnutrition prevalence, we also attempted to identify factors that can predict the significant risk of child malnutrition based on the survey data.

In this study, parental nutrition status (indexed by BMI) was found to be associated with child malnutrition, which was consistent with the results of several previous studies (23, 24). The connection between parental nutrition status and child malnutrition may be due to inherited factors, lifestyle habits, and cultural and social backgrounds. For example, the prevalence of child malnutrition was higher among mothers with lower BMI (25, 26). In light of this significant association, it is of added interest to examine the association of pregnancy BMI, which is not collected in this study, with child malnutrition, as pregnancy BMI can reflect the development of the fetus *in utero* (27).

The findings of this study underscored the importance of parental socioeconomic status, including education and family income, for child malnutrition. Some studies have reported that high family education and high family income are protective factors against child malnutrition (28, 29). By contrast, our findings indicated that high family education and income were significant risk factors in this study. This conflicting observation is explainable. On one hand, with higher family education and income and heavier work pressure, meal time spent with children becomes fewer, which might serve as a possible reason for child malnutrition. On the other hand, the transition from traditional diets to modern diets is characterized by “high sugar, high oil, and less nutrition” which provides a more convenient choice for parents to please their children, especially in high-income households, while commercial foods available on the market do not always deserve the “healthy halo” (30). Moreover, pocket money given by parents is often used to buy unhealthy snacks (31). Therefore, parents are encouraged to accompany their children to eat and to focus on a healthy and balanced diet for their children.

In addition to inherited and environmental factors, dietary factors were also attributable to the occurrence of malnutrition. In this study, we found that dietary habits such as fast food intake frequency, night meals intake frequency, and eating speed were associated with the occurrence of children’s malnutrition. Generally, fast food, which belongs to ultra-processed foods and is devoid of nutrients, can increase the risk of malnutrition, consistent with the results of Khan et al. (32). In addition, frequent night meals and fast eating speed were associated with child malnutrition, and this association was rarely reported in the literature. There is evidence that late dinner eating was linked to low cortisol concentrations and then low appetite during meals (33, 34), and a lower appetite infecting the speed of eating and alimentation could result in childhood undernutrition. Moreover, slow eating may be a symptom of anorexia, a disorder of nutrient absorption. Nevertheless, we cannot exclude the possible impact of different timelines for measurement on the results of this association study. There is no doubt that such poor dietary habits are considered preliminary, and further large, well-designed studies are needed to confirm or refute this finding.

### Limitations

Some limitations should be acknowledged for this study. First, in this study, all participating children were enrolled from Beijing and Tangshan, which are two economically developed cities; hence, extrapolation of our findings to the other areas should be made with caution. Second, data derived from questionnaires filled by parents or caregivers cannot exclude the possibility of recall bias. Third, some socioeconomic data were unavailable for us, such as parental employment. Fourth, the causality of potential risk factors with child malnutrition cannot be addressed due to the cross-sectional design.

## Conclusion

To sum up, we found that approximately 10% of Chinese children aged 3–14 years were in malnutrition status, and seven factors, namely, parental education, family income, fast food intake frequency, night meals intake frequency, eating speed, maternal obesity, and parental obesity, were found to be significant predictors for child malnutrition. For practical reasons, our findings provide an opportunity to understand the risk profiles of child malnutrition and address the utility of screening these factors for primary prevention by changing diets and lifestyles, eventually, preventing the occurrence of child malnutrition.

## Data availability statement

The datasets presented in this article are not readily available because the data includes identifiable data of minors and privacy. Requests to access the de-identified datasets should be directed to zhangzhixin032@163.com.

## Ethics statement

The studies involving humans were approved by The Ethics Committee of China-Japan Friendship Hospital and Beijing University of Chinese Medicine. The studies were conducted in accordance with the local legislation and institutional requirements. Written informed consent for participation in this study was provided by the participants’ legal guardians/next of kin.

## Author contributions

XZ, ZhZ, and WN designed the study. XZ, QW, and ZiZ obtained statutory and ethics approvals. QW and ZhZ contributed to data acquisition. ZG, JW, and WN performed the statistical analysis. XZ and QW wrote the first draft. ZhZ and WN are the study guarantors. All authors contributed to the article and approved the submitted version.

## Footnotes

^1^ https://www.wenjuan.com/
