# Supplementary material for: Prevalence of malnutrition and its associated factors among 18,503 Chinese children aged 3–14 years
Source: Front Nutr. 2023 Dec 11;10:1228799. doi: 10.3389/fnut.2023.1228799 (PMC10750408; doi:10.3389/fnut.2023.1228799)
Supplement: Supplementary file 1 [file Data_Sheet_1.PDF]

Table S1. The assignment of independent variables

**Variables**	**Value assignment**
Gender	Females=0, Males=1
Nationality	Han=1, Minorities=2
Food allergy	No=0, Yes=1
Drug allergy	No=0, Yes=1
Stunting	No=0, Yes=1
Underweight	No=0, Yes=1
Wasting	No=0, Yes=1
Full-term birth (weeks)	Yes=0, No=1
Delivery mode	Vaginal delivery=0, Cesarean section=1
Assisted reproduction	No=0, Yes=1
Twins	No=0, Yes=1
Infancy feeding	Pure breastfeeding=0, Partial breastfeeding=1, Non-breastfeeding=2
Fast food intake frequency	Every day=1, 3-5 times weekly=2, 1-2 times weekly=3, None or once in a while=4
Sweet food intake frequency	Every day=1, 3-5 times weekly=2, 1-2 times weekly=3, None or once in a while=4
Night meals intake frequency	Every day=1, 3-5 times weekly=2, 1-2 times weekly=3, None or once in a while=4
Family education	High school degree or below=0, Bachelor’s degree=1, Master’s degree or above=2
Family income (RMB per year)	<100, 000=0, 100, 000-300, 000=1, >300, 000=2

Table S2. The baseline characteristics of children with stunting, underweight, wasting

Characteristics	Stunting		Underweight		Wasting	
	n=248*	*P*	n=563*	*P*	n=1808*	*P*
**Demographic information**						
Age (years)	5.33 (4.25, 9.67)	<0.001	4.16 (3.83, 4.58)	0.883	5.58 (4.33, 7.33)	<0.001
Males	107 (43.1)	0.01	301 (53.5)	0.235	942 (52.1)	0.583
Nationality		<0.001		0.588		<0.001
Han	215 (86.7)		509 (90.4)		1642 (90.8)	
Minorities	33 (13.3)		54 (9.6)		166 (9.2)	
Height	98 (92, 106.5)	<0.001	105 (101, 109.3)	0.124	115.35 (108, 128)	<0.001
Weight	16 (14.45, 20)	<0.001	9.5 (8.8, 10.5)	<0.001	13 (10.11, 20)	<0.001
BMI	16.62 (14.81, 19)	0.717	8.42 (8.17, 8.98)	<0.001	9.86 (8.4, 12.49)	<0.001
Food allergy	34 (13.7)	0.089	82 (14.6)	0.005	212 (11.7)	0.03
Drug allergy	13 (5.2)	0.5	19 (3.4)	0.708	73 (4.0)	0.736
**Fetal and neonatal factors**						
Birth length	50 (50, 52)	0.096	50 (50, 52)	0.48	50 (50, 52)	0.391
Full-term birth (weeks)	205 (85.4)	0.017	521 (92.9)	0.193	1554 (89.9)	0.913
Delivery mode		0.62		0.21		0.029
Vaginal delivery	118 (47.6)		314 (55.8)		948 (52.4)	
Cesarean section	130 (52.4)		249 (44.2)		860 (47.6)	
Pregnancy order	2 (1, 2)	<0.001	2 (1, 2)	0.621	2 (1, 2)	<0.001
Delivery order	2 (1, 2)	<0.001	1 (1, 2)	0.059	1 (1, 2)	<0.001
Assisted reproduction	8(3.2)	0.099	18 (3.2)	0.128	46 (2.5)	0.017
Twins	6(2.4)	0.988	15 (2.7)	0.395	39 (2.2)	0.469
Infancy feeding		0.123		0.034		0.059
Pure breastfeeding	153 (61.7)		345 (61.3)		1066 (59)	
Partial breastfeeding	70 (28.2)		188 (33.4)		603 (33.4)	
Non-breastfeeding	25 (10.1)		30 (5.3)		139 (7.7)	

Breastfeeding duration (months)	12 (7, 18)	<0.001	12 (8, 18)	0.951	12 (6, 18)	<0.001
Solid food introduction (months)	6 (6, 8)	0.001	6 (6, 6)	0.02	6 (6, 7)	0.713
**Lifestyle-related factors**						
Outdoor activities (hours per day)	1.57 (1, 2.57)	<0.001	1.57 (1, 2.14)	0.001	1.29 (1, 2)	<0.001
Screen time (hours per day)	1.29 (1, 2)	0.005	1 (0.5, 1.29)	<0.001	1 (0.64, 1.57)	<0.001
Fall asleep time (hours per day)	9 (9, 10)	<0.001	9 (9, 10)	<0.001	9 (9, 10)	<0.001
Sleep duration (hours per day)	9.79 (8.57, 10.29)	<0.001	10 (9, 10.93)	0.221	9.29 (8.64, 10)	<0.001
Eating speed (minutes)	18.33 (15, 23.33)	0.082	18.33 (15, 26.67)	0.059	18.33 (15, 25)	<0.001
Fast food intake frequency		<0.001		0.688		<0.001
Every day	26 (10.5)		5 (0.9)		213 (11.8)	
3-5 times weekly	12 (4.8)		5 (0.9)		226 (12.5)	
1-2 times weekly	63 (25.4)		182 (32.3)		483 (26.7)	
None or once in a while	147 (59.3)		371 (65.9)		886 (49.0)	
Sweet food intake frequency		<0.001		0.393		<0.001
Every day	25 (10.1)		48 (8.5)		176 (9.7)	
3-5 times weekly	59 (23.8)		147 (26.1)		572 (31.6)	
1-2 times weekly	128 (51.6)		294 (52.2)		852 (47.1)	
None or once in a while	36 (14.5)		74 (13.2)		208 (11.6)	
Night meals intake frequency		<0.001		0.312		<0.001
Every day	39 (15.7)		58 (10.3)		319 (17.6)	
3-5 times weekly	20 (8.1)		58 (10.3)		266 (14.7)	
1-2 times weekly	51 (20.6)		127 (22.6)		368 (20.4)	
None or once in a while	138 (55.6)		320 (56.8)		855 (47.3)	
**Family-related factors**						
Maternal age	35 (32, 39)	<0.001	36 (33, 39)	<0.001	37 (34, 40)	0.006
Paternal age	34 (31, 37)	<0.001	34 (32, 38)	<0.001	35 (33, 38)	<0.001
Family education		<0.001		<0.001		<0.001
High school degree or below	150 (60.5)		122 (21.7)		555 (30.7)	

Bachelor's degree	87 (35.1)		286 (50.8)		945 (52.3)	
Master's degree or above	11 (4.4)		155 (27.5)		308 (17.0)	
Family income (RMB per year)		<0.001		<0.001		<0.001
<100, 000	148 (59.7)		127 (22.6)		601 (33.2)	
100, 000-300, 000	75 (30.2)		208 (36.9)		683 (37.8)	
>300, 000	25 (10.1)		228 (40.5)		524 (29.0)	
Maternal BMI	22.46 (20.55, 25.24)	0.286	22.04 (20.29, 24.64)	0.384	22.04 (20.2, 24.46)	<0.001
Paternal BMI	24.82 (22.49, 27.76)	0.002	25.25 (22.88, 27.59)	0.224	25.09 (22.82, 27.76)	<0.001

Data are expressed as median (interquartile range) or count (percent). P value was calculated by the rank-sum test or the χ^2 test, where appropriate.

Abbreviations: BMI, body mass index.

[*]Due to the overlap between children with stunting, wasting, and being underweight, the total number of the three components of child malnutrition exceeds 2022, the number of children with malnutrition in this study.

Table S3. Assessment of non-random measurement error for variables under study across different kindergartens or schools.

**Variables**	**ICC**
Age	0.007
Gender	0.005
Delivery mode	0.003
Infancy feeding	0.004
Family education	0.007
Sweet food	0.005
Breastfeeding	0.002
Eating speed	0.006
Night meals	0.005

Abbreviations: ICC, intraclass correlation coefficient.

Table S4. Variance inflation factor between different variables

Variables	VIF	Variables	VIF	Variables	VIF
Age	2.789	Maternal age	3.437	Solid food	1.032
Gender	1.017	Paternal age	3.112	Outdoor activities	1.161
Delivery mode	1.105	Assisted reproduction	1.067	Fall asleep time	1.337
Family income	1.492	Gestational weeks	1.080	Dental caries	1.047
Infancy feeding	1.539	Drug allergy	1.031	Screen time	1.146
Family education	1.737	Food allergy	1.044	Fast food	2.700
Sweet food	1.351	Pregnancy order	1.657	Birth length/5	1.054
Breastfeeding	1.655	Delivery order	1.903	Sleep duration	1.004
Eating speed	1.097	Maternal BMI	1.255	Paternal BMI	1.189
Night meals	1.549				

Abbreviations: VIF, variance inflation factor; BMI, body mass index.

Figure S1. The calibration curve for both basic model and full model

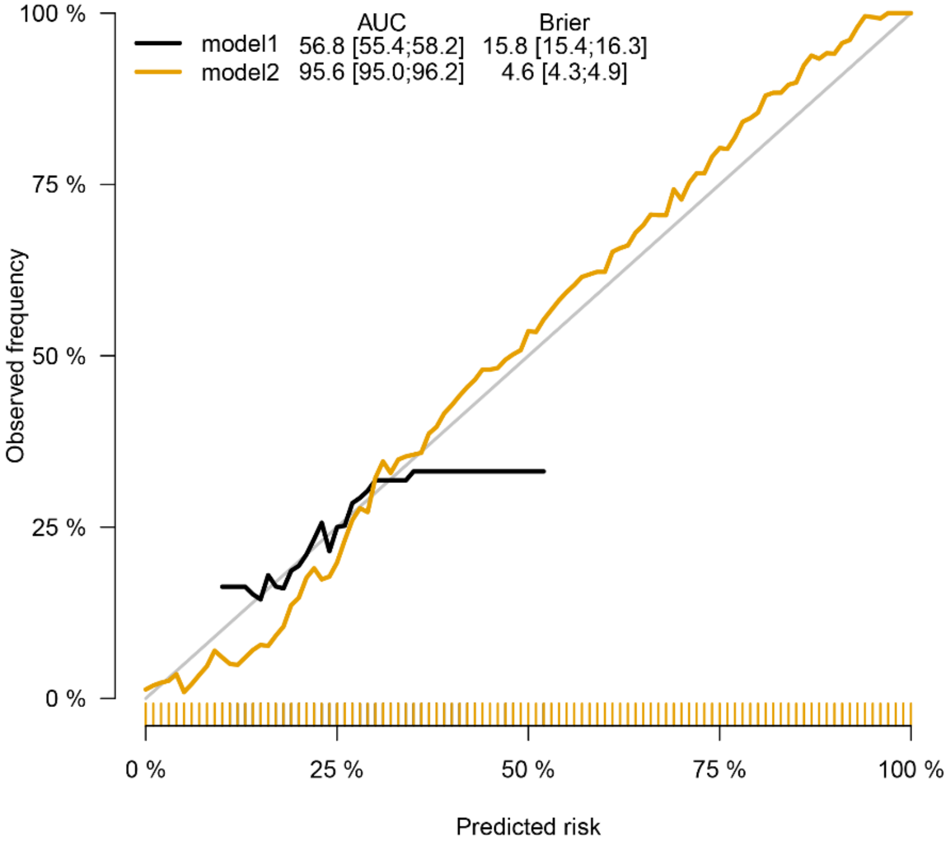

Abbreviations: AUC: area under curve.

The model 1 refers to basic model and the model 2 refers to full model.
